# The Carotid Body a Common Denominator for Cardiovascular and Metabolic Dysfunction?

**DOI:** 10.3389/fphys.2020.01069

**Published:** 2020-08-25

**Authors:** Emilio Badoer

**Affiliations:** School of Health and Biomedical Sciences, Royal Melbourne Institute of Technology (RMIT) University, Melbourne, VIC, Australia

**Keywords:** carotid body, chemoreceptors, hypoxia, hypertension, ventilation, glucose utilization

## Abstract

The carotid body is a highly vascularized organ designed to monitor oxygen levels. Reducing oxygen levels in blood results in increased activity of the carotid body cells and reflex increases in sympathetic nerve activity. A key contributor to elevated sympathetic nerve activity in neurogenic forms of hypertension is enhanced peripheral chemoreceptor activity. Hypertension commonly occurs in metabolic disorders, like obesity. Such metabolic diseases are serious global health problems. Yet, the mechanisms contributing to increased sympathetic nerve activity and hypertension in obesity are not fully understood and a better understanding is urgently required. In this review, we examine the literature that suggests that overactivity of the carotid body may also contribute to metabolic disturbances. The purine ATP is an important chemical mediator influencing the activity of the carotid body and the role of purines in the overactivity of the carotid body is explored. We will conclude with the suggestion that tonic overactivity of the carotid body may be a common denominator that contributes to the hypertension and metabolic dysfunction seen in conditions in which metabolic disease exists such as obesity or insulin resistance induced by high caloric intake. Therapeutic treatment targeting the carotid bodies may be a viable treatment since translation to the clinic could be more easily performed than expected via repurposing antagonists of purinergic receptors currently in clinical practice, and the use of other minimally invasive techniques that reduce the overactivity of the carotid bodies which may be developed for such clinical use.

## Introduction

The carotid body was primarily regarded as a chemoreceptor organ that regulated oxygen levels in the blood. Recent evidence is now showing that in addition to respiratory regulatory functions, the carotid bodies also contribute to hypertension both essential and that associated with chronic hypoxia. Even more recently, evidence is accumulating to suggest that carotid bodies are contributing to glucose utilization and insulin resistance. This review discusses the evidence that the carotid bodies may be a common denominator that contributes to the dysregulation of cardiovascular, respiratory and glucose homeostasis in conditions of metabolic dysfunction such as obesity. We also hypothesize that purinergic receptors of the P2X_3_ subtype may be novel targets that could redress the impaired regulation of the cardiovascular and respiratory systems and glucose utilization in such conditions.

## Carotid Body Anatomy

The carotid body is an oxygen-sensing organ of sympatho-adrenal lineage. It is located near the carotid sinus and the bifurcation of the common carotid artery, but may vary between species (Clarke and de Burgh Daly, 1981). It is a small organ (in humans it is approximately 4 mm in diameter and weighing just over 10 gm) that is highly vascularized and receives its blood supply from a branch of nearby arteries (Clarke and de Burgh Daly, 1981). Sensory fibers, originating from cell bodies in the petrosal ganglion, innervate the oxygen sensing cells in the carotid body.

The carotid body consists primarily of two types of cells known as the glomus or Type I cells and the sustentacular or Type II cells (Biscoe, 1971). Glomus cells are the oxygen sensing cells and are activated by hypoxia through inhibition of oxygen-sensitive potassium channels. Sustentacular cells are glia-like cells that wrap around glomus cells. The functions of the sustentacular cells were believed to be primarily related to supporting glomus cells but recent evidence is appearing to suggest that sustentacular cells can form synaptic-like specializations with glomus cells (Platero-Luengo et al., 2014) which suggests these cells may be playing a key role in the sensory signaling process (Tse et al., 2012; Nurse, 2014). There is also evidence that sustentacular cells are stem cells that can replace/increase the pool of glomus cells in the carotid body (Pardal et al., 2007). These new glomus cells show all the characteristics, and respond, just like glomus cells already present in the carotid body.

## Primary Function of the Carotid Bodies: Protection From Hypoxia

The primary function of the carotid body is in sensing oxygen levels in the blood. In addition to this sensory stimulus, hypercapnia, pH, hypoglycemia, inflammatory mediators, and circulating hormones can influence the afferent information arising from the carotid body through activation of the glomus cells (see later sections for further discussion) (Gonzalez et al., 1994; Nurse, 2005, 2014; Garcia-Fernandez et al., 2007; Zhang M. et al., 2007; Zhang X.J. et al., 2007; Del Rio et al., 2011, 2012; Kumar and Prabhakar, 2012). The mechanisms involved in transmitting low oxygen levels and the activation of the glomus cell are still unclear. Most likely the mitochondrial electron transport chain plays a key part since inhibitors of the mitochondrial electron transport chain mimic the actions of hypoxia. The likely mechanism involves reduced oxygen levels eliciting increased levels of the reduced form of the mitochondrial complex IV which causes a build-up of reactive oxygen species and NADH resulting in a reduction in background K-channel conductance and depolarization of the cell membrane (Buckler, 2015; Fernandez-Aguera et al., 2015; Lopez-Barneo et al., 2016; Prabhakar and Semenza, 2016). An excellent review on the physiology of the carotid body deals with this in much more detail (Ortega-Saenz and Lopez-Barneo, 2019).

What is not in doubt, however, is the essential role of ion channels, particularly potassium channels, of which several have been identified on glomus cells and it is likely that many of these potassium channels contribute to the depolarization of the glomus cells that occurs when oxygen levels fall. Depolarization of the glomus cells induces the opening of calcium channels and the resultant increase in intracellular calcium elicits the exocytotic release of neurotransmitters from granules within the glomus cells.

## The Chemoreceptor Reflex Response to Acute Hypoxia

When the body is subjected to a stimulus that reduces oxygen levels, the chemoreceptors in the carotid body and aortic arch are activated, although, other regions of the body may also contain oxygen sensors (Deane et al., 1975). The most well-studied, of course, are those that emanate from the carotid body and activate afferents in the carotid sinus nerve that relay information to the nucleus tractus solitarius in the medulla oblongata in the brainstem (Finley and Katz, 1992; Finley et al., 1992).

The efferent responses to an acute hypoxic stimulus result in increases in blood pressure and ventilation, responses designed to counteract the initial stimulus. The elevated blood pressure results from rapid increases in sympathetic nerve discharge to the majority of vascular tissues including the renal, adrenal, splanchnic, muscle and cardiac beds (Marshall, 1994; Cao and Morrison, 2001). By contrast, sympathetic discharge to brown adipose tissue is reduced in response to acute hypoxia (Madden and Morrison, 2005).

## Chronic Intermittent Hypoxia

The cardiovascular and ventilatory responses to acute hypoxia are augmented by prior exposure to chronic episodes of intermittent hypoxia (Greenberg et al., 1999; Dick et al., 2007; Marcus et al., 2010; Del Rio et al., 2014). In patients with newly diagnosed obstructive sleep apnea, augmented cardiovascular and respiratory responses to hypoxia are also observed (Narkiewicz et al., 1999). These findings suggest that chronic intermittent hypoxia-induced activation of the chemoreceptor reflex could potentially contribute to pathological cardiovascular and respiratory complications.

Intermittent hypoxia is associated with transient interruption to breathing resulting in transient cycles of oxygen desaturation and re-oxygenation, and can result from obstruction of airway passages and altered respiratory rhythm. Continued intermittent hypoxia chronically over time has deleterious effects as shown by the population cohort that suffers from obstructive sleep apnea (Neubauer, 2001; Wszedybyl-Winklewska et al., 2018). This cohort has been estimated to be 13% of the adult population (Peppard et al., 2013) and the resultant co-morbidities, including cardiovascular dysregulation and hypertension, contribute to poor prognosis and increased mortality (Lavie and Lavie, 2008).

Obstructive sleep apnea is associated with hypertension such that at least 30% of people diagnosed with hypertension suffer from obstructive sleep apnea and this proportion almost triples in those with drug-resistant hypertension (Muxfeldt et al., 2015). Furthermore, the degree of hypertension correlates with the severity of obstructive sleep apnea and male caucasians appear to be most prevalent within the cohort (Hou et al., 2018).

Activation of the carotid bodies during hypoxia induces increased sympathetic nerve activity, and elevated sympathetic outflow is known to contribute to hypertension. Cutting the carotid sinus nerve has been found to reduce blood pressure in spontaneously hypertensive rats suggesting that activated carotid body activity contributes to essential hypertension (McBryde et al., 2017; Niewinski et al., 2017; Paton, 2017). More recently, selective resection of the carotid bodies, and sparing arterial baroreceptor function, also lowered blood pressure in the spontaneously hypertensive rat, strongly implicating overactivity of the carotid bodies as a key contributor to chronic high blood pressure (Pijacka et al., 2018).

Increased sympathetic nerve activity is a contributing cause of the hypertension seen in patients with obstructive sleep apnea (Grassi, 2010; Shell et al., 2016), and in animal models of chronic intermittent hypoxia which develop hypertension (Peng et al., 2014; Takahashi et al., 2018). Overactivity of the carotid bodies may play a key role in the development of hypertension in response to chronic intermittent hypoxia as evidenced by studies showing that ablation of the carotid bodies reduce chronic intermittent hypoxia-induced hypertension (Del Rio et al., 2016).

The overactivation of the carotid bodies induced by chronic intermittent hypoxia and the resultant hypertension may result from increased production of reactive oxygen species (ROS). Following chronic intermittent hypoxia, overexpression of pro-oxidant enzymes like NADPH Oxidase-2 and reduced expression of anti-oxidant enzymes like superoxide dismutase have been detected in the glomus cells and in the brain medullary regions that form part of the central pathways mediating the chemoreceptor reflex [i.e., the nucleus tractus solitarii (NTS) and the rostral ventrolateral medulla (RVLM)] (Peng et al., 2009, 2014). Furthermore, the adrenal gland showed similar changes in the expression of those enzymes suggesting that increased oxidative stress may also occur in the efferent sympathetic outflow and eliciting increased blood pressure. This is supported by the findings that show that scavengers of ROS can reduce the increased blood pressure elicited by chronic intermittent hypoxia in rodents (Iturriaga et al., 2016), and reduce the overexpression of pro-oxidant enzymes and restores the expression of anti-oxidant enzymes in the glomus cells, brainstem, and adrenal gland suggesting a key link between hyperactive carotid bodies, excessive ROS production and elevated sympathetic nerve activity. Strong support for this link has been shown by studies from Prabhakar’s laboratory where the mechanism mediating the increase in ROS and carotid body overactivity has been investigated. Following chronic intermittent hypoxia, that resulted in oxygen levels that simulated sleep apnea, the increased ROS production correlated with DNA methylation that repressed anti-oxidant enzyme genes (Nanduri et al., 2018). Treatment with ROS scavengers prevented the DNA methylation, and ablation of the carotid body prevented the DNA methylation in the NTS and RVLM (Nanduri et al., 2018). Finally, treatment of rats that prevented DNA methylation resulted in normalization of plasma catecholamines and hypertension that occurred following chronic intermittent hypoxia (Nanduri et al., 2018). Taken together the data suggest that DNA methylation and ROS production may be critical in the dysfunction that results from overactivation of the carotid bodies following chronic intermittent hypoxia. Whether the role of DNA methylation contributes to the metabolic disturbances in addition to cardiovascular dysfunction that can accompany carotid body over-activation will be an interesting avenue of research.

## Chronic Intermittent Hypoxia, Obesity, and Metabolic Dysfunction

With chronic intermittent hypoxia, evidence suggests that the effects on glucose homeostasis are detrimental. This is evidenced by increased fasting blood glucose, increased insulin resistance and reduced glucose tolerance as well as impaired pancreatic beta cell function (Polak et al., 2013). The duration of the hypoxia may be correlated to the reduction in insulin sensitivity (Sacramento et al., 2016). Furthermore, although blood glucose may return to normal following cessation of the intermittent hypoxia, continued impairment of glucose tolerance, insulin resistance, and beta cell function can still be observed (Polak et al., 2013), and this may contribute to the detrimental long-term metabolic effects induced by chronic intermittent hypoxia.

Patients suffering from obstructive sleep apnea also show dysfunctional metabolism, including reduced glucose tolerance and elevated risk of type 2 diabetes. Since chronic intermittent hypoxia is a characteristic of obstructive sleep apnea, it would suggest that the chronic intermittent hypoxia may contribute to the detrimental glucose regulation observed in obstructive sleep apnea. There is a close association between obesity and obstructive sleep apnea which may complicate the relationship between dysfunctional glucose metabolism and obstructive sleep apnea. However, even accounting for obesity, obstructive sleep apnea is still an independent risk factor for impaired glucose utilization and type 2 diabetes (Drager et al., 2013). It has also been demonstrated that obese patients with obstructive sleep apnea may have an increased risk of developing metabolic syndrome and higher levels of serum lipids, fasting glucose, and insulin resistance than obese subjects who did not suffer from sleep apnea (Basoglu et al., 2011).

Taken together, the evidence suggests that chronic activation of the carotid bodies may contribute to impaired glucose utilization and thus may be a key factor that links metabolic dysfunction and chronic intermittent hypoxia?

## The Carotid Bodies and Metabolic Dysfunction

Emerging evidence supports the hypothesis that overactivation of the carotid bodies contributes to metabolic dysfunction including the elevated fasting blood glucose and insulin resistance. The mechanisms that link overactivation of the carotid bodies and insulin resistance are not clear but the increase in sympathetic nerve activity and the resultant lipolysis and increased levels of free fatty acids are likely contributors to the insulin resistance (Boden, 2011; Conde et al., 2017).

Denervation of the carotid bodies by cutting the carotid sinus nerve improved insulin sensitivity in mice subjected to chronic intermittent hypoxia suggesting that activity of the carotid bodies can influence glucose homeostasis (Shin et al., 2014). Furthermore, in animal models of diet-induced insulin resistance, there is over activation of the carotid bodies, and when the carotid sinus nerves were cut the sensitivity to insulin was improved and fasting blood glucose was reduced (Ribeiro et al., 2013; Sacramento et al., 2017). More recently, bioelectric modulation to reversibly reduce carotid sinus nerve activity in rats fed a high fat plus high sucrose diet restored insulin sensitivity whilst carotid sinus nerve activity was reduced. However, the impaired insulin sensitivity returned when carotid sinus nerve activity was allowed to return to its abnormally elevated level (Sacramento et al., 2018).

Thus, elevated carotid sinus nerve activity may be an important contributor to the development of insulin resistance and impaired glucose utilization, characteristics of type 2 diabetes. This does not preclude other causes of insulin resistance and impaired glucose tolerance such as the view that hyperinsulinemia is the product of obesity and excess food intake and the concomitant development of insulin resistance (Landsberg and Young, 1978; Reaven, 2004), or the view that increased sympathetic nerve activity decreases glucose uptake and utilization in skeletal muscle resulting in hyperinsulinemia (Masuo et al., 1997; Flaa et al., 2008).

Nonetheless, given the evidence, together with the studies showing that elevated carotid body activity may contribute to the increased sympathetic nerve activity in hypertension; could over-activation of the carotid body be a common factor in dysfunction of glucose utilization and hypertension in conditions in which chronic intermittent hypoxia is a feature?

## Carotid Bodies and Glucose Sensing

Although oxygen sensing is a key function of the carotid bodies, there is evidence indicating that the carotid bodies have additional monitoring capabilities, including responding to glucose levels, which further supports a role of the carotid bodies in glucose homeostasis. In contrast to the pancreatic beta cells in which elevated glucose depolarizes the cells, glomus cells are depolarized by low glucose (Lopez-Barneo, 2003). The mechanisms involved still need to be clarified, however, activation of TRPC3/6 channel subtypes appear to be involved (Garcia-Fernandez et al., 2007). Furthermore, glucose sensing by the glomus cells does not appear to depend on GLUT-2 mediated membrane transport and evidence suggests that metabolites of glucose, independent of hexokinase since this enzyme does not appear to be required in glomus cells, are sensed by the glomus cells (Garcia-Fernandez et al., 2007). Ultimately, the glucose-sensing mechanisms involved in glomus cells involve opening of voltage-dependent calcium channels mediated via the closing of voltage dependent potassium channels and opening of sodium channels (Pardal and Lopez-Barneo, 2002; Lopez-Barneo, 2003).

Activation of the carotid body increases sympathetic nerve activity resulting in increased hepatic glucose release as a counter-regulatory mechanism to counteract hypoglycemia. Such a mechanism also appears to explain the increase in plasma glucose induced in response to intermittent hypoxia seen in animals and humans (Wehrwein et al., 2015; Newhouse et al., 2017). Hypoxia and hypoglycemia have additive effects on the activity of the glomus cells (Pardal and Lopez-Barneo, 2002). Conversely, one would expect that hypoxia and hyperglycemia would have antagonistic actions on glomus cell activity. Since hypoxia and hypoglycemia appear to mediate their excitatory action on glomus cells via independent mechanisms (Garcia-Fernandez et al., 2007), an opportunity may exist to reduce overactivity of the carotid bodies by selectively reducing the influence of hypoxia in conditions in which hypoxia and hyperglycemia are prevalent (i.e., metabolic syndrome and sleep apnea).

## What Are the Potential Transmitters That Can Regulate Carotid Body Sensory Activity

As mentioned earlier, the carotid body is made up of type I glomus cells, sustentacular (type II cells) and the afferent terminals of the petrosal ganglion sensory neurons. Each of these structures are closely apposed to each other and can produce and release potential transmitters and/or express receptors that can be activated. In the following paragraphs we briefly highlight the potentially rich microenvironment that is capable of regulating the afferent sensory responses to hypoxic stimuli. The reader is referred to reviews on this topic for detailed information (Nurse, 2005, 2014; Kumar and Prabhakar, 2012; Nurse and Piskuric, 2013; Zera et al., 2019).

Glomus cells contain many potential chemical transmitters. Acetylcholine, dopamine, histamine, serotonin, adenosine triphosphate (ATP) are amongst the most concentrated within the glomus cells. Acetylcholine and ATP are candidates that appear to be the primary neurotransmitters activating afferent sensory nerves in the carotid body and inhibition of both nicotinic and purinergic receptors, in combination, can prevent the hypoxia-induced responses (Zhang et al., 2000; Zhou et al., 2016). There are species differences, for example, in humans, ACh and ATP also appear to be main mediators of the hypoxic signaling response (Kahlin et al., 2014), just as in rodents and rabbits (Iturriaga and Alcayaga, 2004). However, in the cat dopamine may also play an important role (Iturriaga and Alcayaga, 2004). It should also be noted ACh is inhibitory via muscarinic receptors but excitatory via nicotinic ACh receptors in the rabbit carotid body (Iturriaga and Alcayaga, 2004; Jonsson et al., 2004). Furthermore, transcriptomic studies have compared the human and mouse carotid body transcriptome and shown marked similarities but there were also striking differences in the expression of genes involved in oxygen sensing and cytokine production in the carotid body (Mkrtchian et al., 2012; Kahlin et al., 2014).

Neuropeptides like Substance P, enkephalins, endothelin, and angiotensin II are also present in glomus cells, and so are gaseous neurotransmitters. The glomus cells contain the enzymes required for the synthesis of H_2_S and CO (Peng et al., 2010, 2017, 2019). The generation of H_2_S in glomus cells appears to be a very important mediator in the chemosensory function of the carotid body as highlighted by studies in which the prevention of the generation of H_2_S resulted in the impairment of the respiratory responses induced by hypoxia (Peng et al., 2010, 2017). It has been hypothesized that hypoxia induces a reduction of CO production within the glomus cells and this enables increased production of H_2_S. This appears to contribute to the activation of glomus cells following hypoxic but not anoxic conditions (Peng et al., 2019).

Clearly, such a diverse grouping of neurochemicals involved in influencing the glomus cells and/or the afferent nerve terminals of the sensory fibers, raises the possibility that they have neuromodulatory roles and may contribute to the afferent information depending upon the physiological stimulus.

The activity of glomus cells also may be influenced by the activation of the receptors expressed by the cells. Single cell RNA sequencing techniques have identified several G-protein coupled receptors that are highly expressed on glomus cells (Zhou et al., 2016). These receptors include the olfactory receptor 78, adenosine A2A, purinergic P2Y12, cannabinoid type 1, and pituitary adenylate cyclase-activating peptide type 1. Less frequently found receptors were the angiotensin type, dopamine D2, endothelin A and glutamatergic AMPA and NMDA subtypes. Inhibitory ligand-gated ion channel receptors activated by glycine and GABA were also identified (Zhou et al., 2016).

Afferent sensory terminals in the carotid body are also known to express a broad variety of receptors. These include purinergic P2X, A2A and nicotinic ACh receptors, dopaminergic receptors, serotonergic receptors, neurotrophic receptors (TRK), and TRPV1 receptors, which upon activation can influence afferent activity (Nurse, 2014; Leonard et al., 2018). In addition to potential transmitters produced within the carotid bodies, circulating hormones may also influence carotid body activity, including hormones involved in glucose homeostasis such as insulin and leptin, the renin-angiotensin system and inflammatory mediators.

Insulin receptors have been identified in rat carotid bodies and are functional since insulin increases carotid body activity resulting in increased reflex ventilatory responses and increased blood pressure (Ribeiro et al., 2013). Furthermore, in rats that were insulin resistant due to a high caloric diet, the increase in circulating catecholamines normally observed, was attenuated by denervation of the carotid bodies. The insulin sensitivity was restored to normal and the hypertension seen in rats on the hypercaloric diet was also reduced following the denervation of the carotid bodies (Ribeiro et al., 2013). These experiments provide strong support for a physiological role of the carotid bodies in glucose homeostasis and suggest that circulating insulin can activate the carotid bodies. In humans, the physiological role of insulin on carotid body function still needs to be clarified, Infusions of insulin have been shown to increase muscle sympathetic nerve activity, but this could not be attenuated by low dose dopamine and/or hyperoxia suggesting acute insulin infusion does not affect carotid body function (Limberg et al., 2020). However, in chronic conditions like diabetes, insulin may well have an important role in glucose homeostasis mediated by the carotid bodies (Vera-Cruz et al., 2015).

Leptin receptors have also been identified on glomus cells and their activation by leptin results in increased glomus cell activity (Porzionato et al., 2011; Messenger and Ciriello, 2013). Interestingly, leptin is expressed within glomus cells suggesting that both locally produced and systemic leptin may influence carotid body activity. Furthermore, the leptin receptors are downregulated by intermittent hypoxia but the expression of leptin itself in the carotid body is upregulated by intermittent hypoxia (Messenger and Ciriello, 2013), suggesting a complex inter-relationship between intermittent hypoxia and leptin on carotid body activity. This issue has been addressed by an interesting recent study that found up to 74% of glomus cells in mice expressed the leptin receptor and that the hypoxia-induced increase in the activity of the carotid sinus nerve was enhanced by leptin administration (Caballero-Eraso et al., 2019). Furthermore, this study also showed that in mice deficient in the leptin receptor (i.e., *db/db* mice), re-introduction of the leptin receptor in the carotid bodies of these mice increased minute volume and the ventilatory response to hypoxia (Caballero-Eraso et al., 2019), confirming that leptin is playing a key physiological role in carotid body function.

Leptin’s role in carotid body function does not appear to be restricted to respiratory regulation. Leptin administration is well-known to induce increases in blood pressure, and in hypertension associated with obesity, it has been long recognized that the cardiovascular responses to leptin are not reduced which is in stark contrast to the effects on dietary intake which were diminished compared to lean controls. Such observations led to the concept of “selective leptin resistance” (Prior et al., 2010; Mark, 2013).

A recent exciting study has now highlighted a key role for leptin in the carotid body (Shin et al., 2019). In that study, peripheral leptin administration induced increases in blood pressure in lean mice and the effect was prevented by carotid body denervation. The hypertensive response initiated by the activation of leptin receptors was mediated by TRPM7 calcium channels. Furthermore, overexpression of the leptin receptor selectively in the carotid body of leptin receptor-deficient mice enhanced TRPM7 gene expression and induced hypertension (Shin et al., 2019). Thus, taken together, the findings suggest that leptin acting within the carotid body is playing a major role in the cardiovascular and respiratory function. The contribution of leptin within the carotid body to glucose homeostasis clearly needs investigation and potentially would support the hypothesis linking the carotid body activity as a common mediator regulating cardiovascular, respiratory and glucose homeostasis. The role of TRPM7 channels would be an exciting focus.

The peptide hormone, angiotensin, can also influence carotid body activity. The angiotensin II type I receptor (ATIR) is found on glomus cells in the carotid body and angiotensin II predominantly stimulates the carotid body as determined from electrophysiological recordings of the carotid sinus nerve (Allen, 1998). The expression of AT1R in the carotid body is increased by chronic intermittent hypoxia and this may contribute to the increase in sympathetic nerve activity elicited by chronic intermittent hypoxia (Marcus et al., 2010; Takahashi et al., 2018). Furthermore, the enhanced activation of lumbar sympathetic nerve activity in rats following chronic intermittent hypoxia was prevented with treatment using the ATIR antagonist, losartan, suggesting angiotensin II activation of the carotid body played an important role in sympathetic nerve activity responses induced by chronic intermittent hypoxia (Marcus et al., 2010).

In obesity, the renin-angiotensin-aldosterone system may take on more importance since adipose cells can produce angiotensinogen (Cassis et al., 1988), and overexpression of angiotensinogen in adipose tissue can contribute to hypertension and adipose tissue development (Massiera et al., 2001). In humans, obstructive sleep apnea has been reported to increase the activity of the renin-angiotensin-aldosterone system (Goodfriend and Calhoun, 2004). Thus, angiotensin II may be an important link between obesity and chronic intermittent hypoxia and overactivity of the carotid body.

Evidence also suggests that inflammatory mediators within the chemosensory pathways are key mediators of cardiorespiratory dysfunction. Studies using human carotid bodies (taken from patients undergoing surgical procedures for head and neck tumors) show that hypoxia can induce the release of cytokines (Kahlin et al., 2014). Furthermore, chronic intermittent hypoxia in rats induces increased production of pro-inflammatory cytokines in the carotid body and in the nucleus tractus solitarii (Del Rio et al., 2011). This increased production of pro-inflammatory cytokines and the resultant cardiorespiratory responses can be prevented by pretreatment with the non-steroidal anti-inflammatory drug, ibuprofen (Del Rio et al., 2012). Since obesity is associated with abnormally elevated sympathetic nerve activity, elevated levels of cytokines, adipokines and obstructive sleep apnea (Lambert et al., 2010; Armitage et al., 2012), and the observation that intermittent hypoxia has been reported to induce inflammation in adipose tissue (Poulain et al., 2017), the evidence suggests that increased levels of pro-inflammatory cytokines within the peripheral and central components of the chemosensory pathways contribute to the cardiovascular and respiratory dysfunction seen in conditions like obesity that involve chronic intermittent hypoxia.

## Is There a Role for Purines in the Carotid Body in the Regulation of Glucose Homeostasis?

To date, there have been no reports directly investigating whether inhibition of purinergic function can influence glucose homeostasis. However, the following evidence provides circumstantial evidence in support of such a hypothesis.

The release of ATP in response to hypoxia is well-recognized. ATP and adenosine (which can be produced through the metabolism of ATP) activate sensory afferent neurons in the carotid body through P2X and A2A purinergic receptors (Zhang and Nurse, 2004; Conde et al., 2006), which results in increased activity of the carotid sinus nerve. Inhibition of P2X2/3 and A2A purinergic receptors can reduce carotid sinus nerve activity, and the resultant reflex ventilatory responses (Zhang et al., 2000, 2018; Conde et al., 2006). Thus, activation of the carotid body in response to hypoxia involves purinergic receptor activation.

In addition to mediating reflex respiratory function, the increased carotid sinus nerve activity induced by carotid body activation also results in increased sympathetic nerve activity and blood pressure, as indicated earlier. There is good evidence indicating a role of P2X_3_ receptors in mediating this cardiovascular reflex response. In an extensive study by Pijacka and colleagues, they showed that spontaneously hypertensive rats have a hyper-reflexive response to acute stimulation of the carotid body chemoreceptors and this is associated with upregulation of the P2X_3_ receptors in the carotid body (note P2X2 and P2X_3_ receptors can heterodimerize to form functional purinergic P2X2/3 receptors). Furthermore, antagonism of P2X_3_ receptors with a selective antagonist reduced sympathetic nerve activity and blood pressure in the spontaneously hypertensive rat (Pijacka et al., 2016). There was no effect in the normotensive control rats. This led the authors to suggest that using the P2X_3_ receptor antagonist may reduce overactivity of the carotid body and this was supported by the finding that petrosal ganglion neurons (cell bodies of the carotid body afferent fibers) from Spontaneously Hypertensive rats were sensitized to purinergic receptor activation but this was not observed in normotensive Wistar rats. Furthermore, the activity of the petrosal ganglion neurons from Spontaneously Hypertensive rats was normalized by P2X_3_ receptor antagonism (Pijacka et al., 2016). Taken together, the findings suggest that normal function of the carotid body chemoreceptors may not be markedly affected by administration of purinergic P2X_3_ receptor antagonists but overactivity of those chemoreceptors may be attenuated.

Thus, respiratory and cardiovascular function can be influenced by purinergic P2X_3_ receptor antagonism, whether regulation of glucose homeostasis can be added to this action has not been reported to date. However, the circumstantial evidence available points to such a possibility. Very interesting work emanating from Conde and colleagues has shown that there is increased sympathetic nerve activity in rats fed high caloric diets that have hypertension, hyperinsulinemia and hyperglycemia. In these rats, carotid body denervation reduced sympathetic nerve activity, blood pressure and restored insulin sensitivity and glucose utilization (Ribeiro et al., 2013). Externally applied, bioelectric modulation has recently been used to reversibly reduce carotid sinus nerve activity in rats fed high caloric diets and restore insulin sensitivity (Sacramento et al., 2018). Whether purinergic P2X_3_ receptor antagonism could induce similar positive effects on insulin sensitivity needs to be investigated. Interestingly, a positive finding may be readily translatable to clinical situations since P2X_3_ receptor antagonists have been developed and one is currently in phase III trials for persistent cough (ClinicalTrials.gov Identifier: NCT03449134) and may be useful in other hyper-reflexive states (Richards et al., 2019).

## Clinical Translation

Unilateral and bilateral carotid body removal have been utilized in small clinical studies of patients with drug-resistant hypertension and in patients with low ejection fraction heart failure. In about half of the hypertensive patients, blood pressure was reduced even up to 12 months post-unilateral carotid body resection. The responders were those that had enhanced ventilatory responses to chemoreceptor stimulation prior to the procedure (Narkiewicz et al., 2016). In the heart failure patients, the quality of life was improved but given the size of the cohort, much more work is needed, particularly since bilateral carotid body resection was often associated with poorer nocturnal oxygen saturation content suggesting an increase in sleep apnea (Niewinski et al., 2017). Therefore, bilateral carotid body resection may not be an entirely safe viable therapeutic option for most patients. However, modulating the activity of the carotid sinus nerve by targeting specific receptors (e.g., purinergic P2X_3_) or ion channels (e.g., TRPM7) could prove to be a useful therapeutic intervention. Bioelectric modulation of neuronal activity is another relatively non-invasive mechanism that could be explored (Iturriaga et al., 2016).

## Summary and Conclusion

**FIGURE 1:**
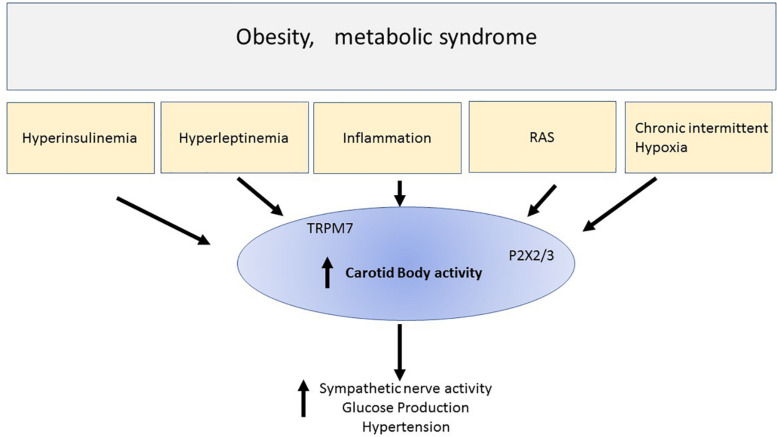
This simple schematic highlights the possibility that chronic intermittent hypoxia and hyper-insulinemia and hyperleptinemia inflammation and elevated renin -angiotensin aldosterone system (RAS) that may be observed in metabolic conditions like obesity or metabolic syndrome, activate the carotid bodies. The result includes increase sympathetic nerve activity, hyperglycemia, and hypertension. Activation of the carotid body can result from many chemicals and receptors that were discussed in the text. The schematic highlights ATP released from glomus cells and acting on purinergic receptors (P2X_3_) which we suggest is particularly important in contributing to the activation of the afferent pathways initiated by chronic intermittent hypoxia. We also highlight new findings suggesting that stimulation of leptin receptors activates TRPM7 ion channels. Thus, overactivity of the carotid body may be a common denominator involved in the cardiovascular and metabolic disturbances seen in metabolic disorders. Reducing the overactivity of carotid bodies, for example using P2X2/3 antagonists which are in clinical practice, may prove to be a novel therapeutic treatment regime, or developing non-invasive bioelectric modulation techniques may prove useful therapeutic strategies.

The carotid bodies are small organs that are exquisitely sensitive to the level of oxygen in the blood and initiate a response to hypoxia that includes increased sympathetic nerve activity, increased blood pressure and increased ventilation and increased glucose production due to catecholaminergic actions on the liver. Chronic intermittent hypoxia is associated with obesity and overactivity of the carotid bodies has been described in this metabolic condition (Figure 1). Thus, reducing this overactivity may be beneficial for the cardiovascular, respiratory and hyperglycemia present in obesity and other conditions of metabolic dysfunction. There is evidence supporting this. Therefore, the carotid bodies may be a common link and thus an attractive potential target to tackle the cardiorespiratory and metabolic dysfunction that are observed in obesity and metabolic syndrome. Potential approaches to therapy include the development of antagonists to TRPM7 channels and P2X_3_ receptors which may contribute to the increase in sympathetic nerve activity in response to hypoxia (Figure 1). Antagonists to P2X_3_ receptors are already in late clinical trials (for persistent cough) which may make translation to other clinical conditions more practicable. Bioelectric modulation, which is a relatively non-invasive technique to lower carotid sinus nerve activity, may also find translation into the clinical setting more readily.

## Author Contributions

EB wrote the review and designed the figure.

## Conflict of Interest

The authors declare that the research was conducted in the absence of any commercial or financial relationships that could be construed as a potential conflict of interest.
